# Protocol for evaluation of the cost-effectiveness of ePrescribing systems and candidate prototype for other related health information technologies

**DOI:** 10.1186/1472-6963-14-314

**Published:** 2014-07-19

**Authors:** Richard J Lilford, Alan J Girling, Aziz Sheikh, Jamie J Coleman, Peter J Chilton, Samantha L Burn, David J Jenkinson, Laurence Blake, Karla Hemming

**Affiliations:** 1 Warwick Medical School, University of Warwick, Coventry CV4 7AL, UK; 2 College of Medical and Dental Sciences, University of Birmingham, Edgbaston, West Midlands B15 2TT, UK; 3 eHealth Research Group, Centre for Population Health Sciences, The University of Edinburgh, Edinburgh EH8 9AD, UK; 4 Division of General Internal Medicine and Primary Care, Brigham and Women’s Hospital/Harvard Medical School, Boston, MA 02115, USA

**Keywords:** ePrescribing, Health information technology, Cost-effectiveness, Adverse events, Bayesian elicitation, Probability densities

## Abstract

**Background:**

This protocol concerns the assessment of cost-effectiveness of hospital health information technology (HIT) in four hospitals. Two of these hospitals are acquiring ePrescribing systems incorporating extensive decision support, while the other two will implement systems incorporating more basic clinical algorithms. Implementation of an ePrescribing system will have diffuse effects over myriad clinical processes, so the protocol has to deal with a large amount of information collected at various ‘levels’ across the system.

**Methods/Design:**

The method we propose is use of Bayesian ideas as a philosophical guide.

Assessment of cost-effectiveness requires a number of parameters in order to measure incremental cost utility or benefit – the effectiveness of the intervention in reducing frequency of preventable adverse events; utilities for these adverse events; costs of HIT systems; and cost consequences of adverse events averted. There is no single end-point that adequately and unproblematically captures the effectiveness of the intervention; we therefore plan to observe changes in error rates and adverse events in four error categories (death, permanent disability, moderate disability, minimal effect). For each category we will elicit and pool subjective probability densities from experts for reductions in adverse events, resulting from deployment of the intervention in a hospital with extensive decision support. The experts will have been briefed with quantitative and qualitative data from the study and external data sources prior to elicitation. Following this, there will be a process of deliberative dialogues so that experts can “re-calibrate” their subjective probability estimates. The consolidated densities assembled from the repeat elicitation exercise will then be used to populate a health economic model, along with salient utilities. The credible limits from these densities can define thresholds for sensitivity analyses.

**Discussion:**

The protocol we present here was designed for evaluation of ePrescribing systems. However, the methodology we propose could be used whenever research cannot provide a direct and unbiased measure of comparative effectiveness.

## Background

### Provenance

This protocol concerns the assessment of cost-effectiveness of hospital health information technology (HIT). The cost-effectiveness analysis forms part of a National Institute for Health Research (NIHR) funded research programme to evaluate the implementation, adoption, effectiveness and cost-effectiveness of ePrescribing systems as they are introduced into a sample of hospitals in England (RP-PG-1209-10099). Four hospitals will be studied – before, during, and after implementation of an ePrescribing system, as described in the application for funding (RP-PG-1209-10099) [1-4]. Two hospitals are acquiring systems with extensive decision support, while the other two will implement systems incorporating only the most basic clinical algorithms. Three types of data will be collected from each site:

1. Qualitative data on the acceptability and adoption of the system;

2. Quantitative data on prescribing safety;

3. Cost data.

In this paper, we describe the protocol for the cost-effectiveness analysis that will follow data collection. For reasons that have been described in a previous paper [5], cost-effectiveness analysis of large scale service delivery interventions raises issues that are not part of standard Health Technology Assessment (HTA). We now describe some of these issues in more detail.

### Issues in evaluation of large scale service changes

#### Diffuse impact of generic health information technology interventions

**Figure 1:**
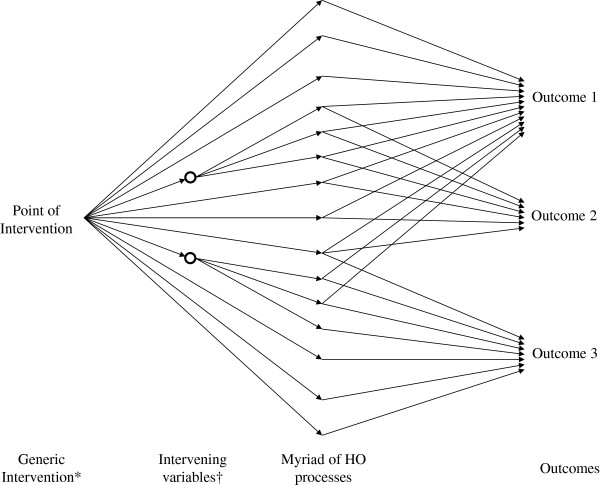
**Representation of the widespread effects of a generic intervention.** *First as intended and then as actually implemented. †Sometimes referred to as organisation level outcomes to include morale, staff attitude, knowledge, effect on patient flows etc.

Implementation of an ePrescribing *system* is an example of a generic intervention with diffuse effects, spanning out over myriad clinical processes [5], in contrast with more targeted interventions focussed on a limited number of end-points. This crucial distinction is represented diagrammatically in Figure 1. Some applications of HIT have narrow focus – mobile phone-based decision support to improve compliance with asthma treatment, for example [6] – and can be considered as examples of targeted service interventions. On the other hand, a comprehensive ePrescribing *system* has many of the features of a generic service intervention. It has a potential impact on work patterns (at the system level) and it may affect a large number of clinical processes (e.g. prescriptions) and contingent outcomes (e.g. preventable adverse events) at the clinical level. It is important to note that each adverse event may be affected to a different degree by the intervention and will be associated with a particular utility. This is in contrast to typical HTA, which may have an effect on one, or a limited number of, outcomes. The protocol thus has to deal with a large amount of information collected at the system, clinical process and outcome ‘levels’.

#### Lack of contemporaneous controls

This study, in keeping with many in the service delivery/quality improvement literature, is based on a before and after design. A preferable controlled before and after design [7] (or randomised comparison) was not possible within the funding envelope. The study therefore cannot control for general temporal trends and is also subject to selection effects given the non-experimental design. The protocol thus needs to find a way to accommodate the possibility of bias in estimates of parameters used to populate the health economic model.

#### Integrating study results with evidence external to the index study

Given the above uncertainties, decision makers will want to ensure that parameters used in the estimation of cost-effectiveness take account of evidence from the large literature on HIT systems [8,9]. This cannot be achieved by standard meta-analysis given the highly variegated nature of the salient literature.

### Confronting the issues – epistemology of large-scale service changes

Elsewhere we have suggested that generic service, and many policy, interventions cannot be evaluated solely by direct parameter estimates that have been so successful for the evaluation of clinical treatments and for targeted service interventions [10]. The framework outlined above provides a way forward in circumstances where ‘knock-down’ evidence is elusive. It provides a ‘half-way house’ between fruitless striving for a clear cut quantitative ‘answer’ and reverting to a completely unquantified ‘interpretivist’ [11] or even ‘realist’ approach. We propose use of Bayesian ideas as a philosophical guide (as proposed by Howson and Urbach) [12] rather than a mathematical method to update a prior probability density. This issue is explored further in the discussion.

The scientific method can thus be conceptualised as the process by which data are collected and analysed so as to inform a degree of belief concerning the parameter(s) of interest [13]. The data concerned may be of various types. These diverse data types are assembled to inform a probabilistic judgment.

The intellectual model we propose has the following features:

1. Its epistemology is Bayesian, treating probability as a degree of belief.

2. Quantitative study data are not used as direct parameter estimates for use in models, but as information to inform subjective estimates of effectiveness.

3. Qualitative study data will also contribute to the subjective estimates of effectiveness.

4. Subjective probability densities will be elicited from groups of experts exposed to the above quantitative and qualitative data, and also data from studies external to the index study.

5. The densities will be pooled across experts for use in health economic models (both for the base case and to describe thresholds for sensitivity analyses).

In summary, we will assemble both quantitative and qualitative data, from different sources, to triangulate any evidence of effectiveness or lack of effectiveness, and establish parameters that summarise evidence of effectiveness [10].

## Methods/Design

### Overview of cost-effectiveness model

Evaluation of cost-effectiveness will proceed as follows [14]:

1. Evaluate effectiveness in reducing the frequency of preventable adverse events;

2. Assign utilities for these adverse events;

3. Calculation of expected health benefit;

4. Determine costs (fixed and recurrent) of procuring, implementing, operating and maintaining HIT systems and model the cost consequences of adverse events averted;

5. Calculation of cost-effectiveness.

**Figure 2:**
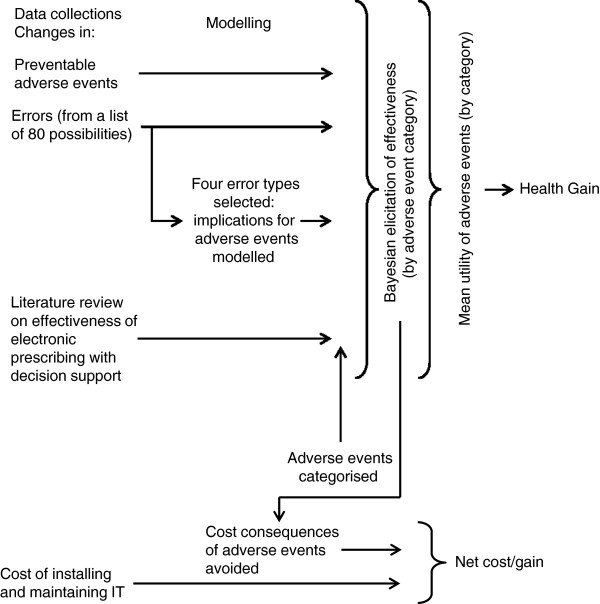
Framework for the evaluation.

The first two quantities (effectiveness in reducing adverse events, and utilities) are used to calculate health benefit (assuming that this cannot be captured directly through a quality of life measurement – see below). Component 4 allows *net* costs to be estimated. Costs and benefits can then be consolidated in a measure of incremental cost utility or cost benefit. An overall framework for our proposed evaluation is given in Figure 2. The perspective of the evaluation is that of the health services, at least in the first instance – a point to which we return.

### Evaluation of effectiveness

#### Consideration of quantitative end-points

There are four (non-exclusive) end-points that may be used in measurement relating to effectiveness:

1. Generic quality of life;

2. Adverse event rates (including mortality);

3. Error rates;

4. Triggers (for errors or adverse events).

None of the above end-points are unproblematic. We now discuss each to determine which are more suitable in the context of this study.

##### Generic quality of life

A generic measurement of quality of life, using a measurement tool such as the SF36 [15], is an attractive option because such a measurement (when combined with death) consolidates all the various adverse events that the intervention is designed to prevent. This end-point thus gets around the problem that each adverse event has its own utility and may be affected differentially by the intervention. The problem, however, is that prescribing errors make only a very small contribution to generic quality of life since less than 1% of patients suffer a preventable medication-related adverse event during a single hospital stay and the majority, as we shall see later, are minor and short-lived [16]. In short, any ‘signal’ would be lost in ‘noise’; a false null result would be likely even if worthwhile improvement had occurred.

##### Medication-related adverse events

Again, the value of this end-point is limited by issues of statistical power as a result of the ‘ceiling’ for improvement in preventable events of approximately one percentage point [17], as mentioned above. The sample size calculations in Table 1 show that a very large number of cases would have to be examined to avoid a high risk of a false null result in detecting preventable adverse events. Detecting medication-related adverse events with adequate specificity for use in a comparative study requires direct observation or case-note review, meaning it would be impossible (or at least ruinously expensive) to conduct an adequately powered study on this basis.

**Table 1:** Sample size calculations for detection of reductions in adverse event rates

**Risk ratio**	**Power (%)**	**Sample size (total)**
0.6	80	16,556
0.7	80	30,716
0.8	80	71,988
0.6	90	40,676
0.7	90	41,294
0.8	90	95,702

Sample size calculations for detection of reductions in preventable adverse event rates in a simple comparison of two equally sized groups of patients – assumes a two-tailed alpha of 0.05 (without continuity correction) and a control probability of 1%. Results from STATA v12.0.

##### Prescribing error rates

Prescribing errors are much more common than error-related (i.e. avoidable) adverse events; the baseline error rate is about 5% [18], and hypothesised reductions in these errors of 30% (two percentage points) or more are in line with those found in the literature [8,9]. This end-point therefore yields more manageable sample size requirements (Table 2). Samples sufficient to detect a 30% improvement with 80% power are feasible under the funding envelope of the study. However, this end-point is far from perfect because:

**Table 2:** Sample size calculations for detection of reductions in error rate

**Risk ratio**	**Power (%)**	**Sample size (total)**
0.6	80	3,210
0.7	80	5,940*
0.8	80	13,888
0.6	90	4,230
0.7	90	7,862
0.8	90	18,456

*Similar to proposed sample in this study.

Sample size calculations for detection of reductions in error rate; baseline error rate 5% and other assumptions, as in Table 1.

1. Errors are surrogates for adverse events. It is therefore necessary, in any cost-effectiveness analysis, to infer adverse event rates from error rates – a step that introduces further uncertainty.

2. Error rates are associated with considerable measurement error [19], and detection can be affected by learning effects, fatigue [20], and conceivably also by use of a computer.

3. The more serious an error, the less likely it is to be perpetrated [21], and so a study based on a limited sample is likely to underestimate effects of an intervention on rare, but egregious, errors.

##### Trigger tool methods

Triggers are based on evidence suggesting that a preventable adverse event might have occurred (e.g. administration of vitamin K or anti-narcotics to reverse a putative overdose of warfarin or morphine respectively). The triggers are selected on the basis that they can be easily ascertained from existing data systems – it is easy to search the pharmacy database for use of the above antidotes, for example. Such triggers can be useful in quality improvement programmes where the IT system remains stable over a period where a non IT-based safety intervention is introduced [22]. They are likely to yield a biased result, however, when the IT system is both the intervention of interest and used in collection of end-point data. Furthermore, triggers are not only non-specific, but insensitive [23]. This is because only a small proportion of all medication-related adverse events show up on a trigger tool system.

#### Selection of quantitative end-points

It can be seen from the above analysis that there is no single end-point that adequately and unproblematically captures the effectiveness of the complex intervention that we have been commissioned to study. Following discussion with the programme Steering Group we decided to reject two of the above four possible end-points. Trigger tools were rejected on the grounds that while they are useful in quality control systems within a stable platform, they are likely to be a highly unreliable (biased and imprecise) tool for scientific measurement of the effectiveness of a HIT system. Generic quality of life questionnaires were rejected on the grounds that they could not detect improvement among the small proportion of patients that suffer an avoidable medication-related adverse event.

We will measure error rates and adverse events as the ‘least bad’ options in this study. Error rates will be measured as described in detail elsewhere [24]. In brief, a specified list of 80 errors with potentially serious consequences has been identified by a consensus technique. [25] These errors are reasonably common and by concentrating on a limited number we believe we can identify them with high sensitivity irrespective of the ‘platform’ in use – i.e. irrespective of whether the computer system has been deployed. To mitigate measurement error, observers will be trained, and to reduce the effect of prescribing systems on measurement the reviewers will be on site with access to all prescribing information, whether held on computer or recorded on paper. In this way we plan to make the data collection task as independent as possible from the intervention. We intend to identify errors by examining every prescription within a sample of consecutive patients, as used in many other studies [18].

The observers will also record adverse events that come to light during the study. Each patient case note will be reviewed for adverse events, which will then be examined in detail to determine whether, on the balance of probabilities, they were preventable.

#### Illustrative modelling of adverse events from errors

Errors are important only insofar as they portend adverse events. In order to illustrate the pathway between errors and preventable adverse events, we will model expected reductions in adverse events from (any) reductions in error rates. Since doing so for all 80 errors on the above list would be a laborious and expensive process, we shall do so for exemplars across four error classes – drug interactions, allergy, dose error and contra-indications. Within these classes we have selected errors for which information to populate causal models is available in the literature – a point taken further in the discussion. Further details on this method are given in Additional file 1. Patients are exposed to the risk of error and hence of an adverse event when attending hospital and receiving a prescription. As these are mainly one-off prescriptions, decision trees will be used to model the risk of adverse events. For each of the illustrative errors chosen, the probability of contingent adverse events will be modelled on the basis of information in the literature. Markov chains will be used when one adverse event may lead to another – for example deep venous thrombosis that may lead to pulmonary embolism that, in turn, may lead to death. In this way, we will compute the headroom for reductions in adverse event rates related to certain specific errors, i.e. the reduction in adverse event rates that would be expected if the causal errors could be eliminated. The results will be used to assist expert judgement within the elicitation of subjective probability densities, as described below. Our expectation is that the results of this modelling exercise, with respect to just four errors, will help experts to mentally ‘calibrate’ their subjective probability estimates, with respect to error in general. More specifically, we think that it will mitigate heuristic biases, such as over-confidence and anchoring, itemised by Kadane and Wolfson [26].

#### Classifying adverse events

As stated above, the purpose of the elicitation exercise is to estimate reductions in adverse events. We will have to deal with the fact that there are a very large number of different preventable adverse events. It cannot be assumed that an intervention will affect all events equally. Moreover, each event is associated with its own mean utility. Ascribing a single probability and utility to cover all adverse events is too crude. On the other hand, ascribing a probability and utility to each and every event detected in the study or inferred from errors would be a logistically taxing process and would omit certain rare, but notorious, events such as daily rather than weekly methotrexate administration. Our approach to this problem builds on a previous study by our group, where adverse events were classified according to severity and duration [14]. Classification systems that have been described in the literature are explicated in Table 3.

**Table 3:** Classification systems for adverse events, with prevalence figures (proportion of total adverse events in given category)

**Forster et al. **[27]		**Brennan et al. **[17]		**Hoonhout et al. **[28]		**Yao et al. **[14]	
**Event category**	**Proportion in category**	**Event category**	**Proportion in category**	**Event category**	**Proportion in category**	**Event category**	**Proportion in category**
Death	0	Death	0.136	Death	0.078	Death	0.05
Permanent disability	0.03	Permanent impairment, >50% disability	0.026	Permanent disability	0.047	Permanent impairment, >50% disability	0.02
		Permanent impairment, ≤50% disability	0.039			Permanent impairment, ≤50% disability	0.03
Readmission	0.21	Moderate impairment, recovery >6 months	0.028	Moderate disability	0.617	Moderate impairment, recovery >6 months	0.10
A&E visit	0.11	Moderate impairment, recovery 1–6 months	0.137			Moderate impairment, recovery 1–6 months	0.30
Physician visit	0.14	Minimal impairment, recovery <1 month	0.634	Minimal effect	0.257	Minimal impairment, recovery <1 month	0.50
No extra use of health service	0.51						

We shall use the four category system (i.e. dead, permanent disability, moderate disability, minimal effect) proposed by Hoonhout and colleagues [28]. We have selected this system for two reasons. First, it has the smallest number of categories, and will therefore be the least tedious to implement when probabilities and utilities are elicited. Second, this is the only system for which the costs associated with preventable adverse events in each category are available (Table 4). In subsequent calculations we will make use of the probability of each category of adverse event arising as a result of treatment given in hospital. This is given by the product of the proportions of adverse events in each category (Table 4) and the prevalence of all preventable adverse events (i.e. 0.01 [1%] as referenced above).

**Table 4:** Classification of preventable adverse events that we propose to use in this study*

**State**	**Proportion**	**Utility**	**Mean duration, L (years)**	**Cost, 2009 (€)**	**Comments**	**Example**
Death	0.078	0	3	3,831	Duration here is expected mean survival without the event, as estimated as weighted average from Zegers et al. [29]	Vincristine administered by intrathecal route.
Permanent disability	0.047	To be determined	6	6,649	Costs exclude long-term care. No data on mean duration, but a given adverse event is more likely to be fatal in an older person, so mean survival assumed to be a little longer than life years lost in those who died.	Haemorrhagic stroke in patient prescribed warfarin and macrolide antibiotics.
Moderate disability	0.617	To be determined	0.2	5,973	Duration ≤6 months in 70% of cases (Baker et al. [30])	Pulmonary embolism in large patient given standard (inadequate) dose of heparin.
Minimal effect	0.257	To be determined	0.05	2,979		Transient urticarial rash in known allergic patient given penicillin.

*Based on Hoonhout et al. [28]

#### Qualitative data

As stated in the introduction, the full evaluative study includes a qualitative component. As discussed in the section on epistemology, the qualitative data are used to inform Bayesian elicitation alongside quantitative data. In the case of ePrescribing systems, organisation-level data, such as the success of implementation and staff attitude, have a bearing on effectiveness [1-4]. A qualitative finding that these elements are positive would reinforce a statistical observation that medication errors had been reduced, and yet this finding would be difficult to incorporate into an objective analysis. Our approach provides a way out of this conundrum by providing quantitative parameter estimates (for use in a decision model) that effectively combine qualitative and quantitative information through the elicitation of probability densities.

#### Eliciting subjective probability densities

**Figure 3:**
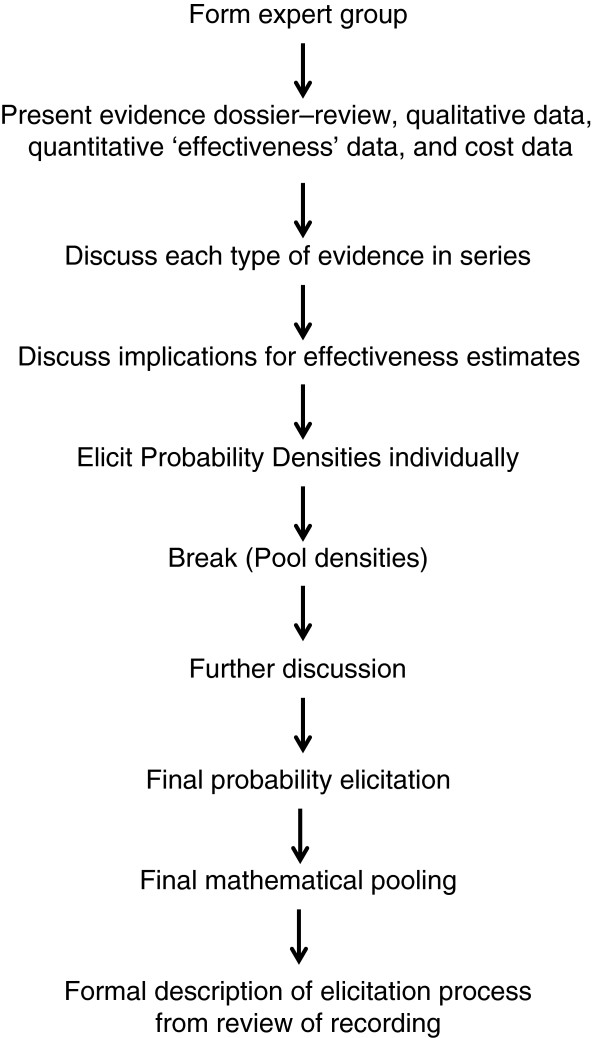
Sequence of events for elicitation of Bayesian probability densities.

We propose to elicit subjective probability densities for an effectiveness parameter for each of the Hoonhout sub-groups. As discussed before, we are not adhering to the usual paradigm, whereby a prior is elicited and then updated in a statistical manner by means of direct comparative data. Rather, we wish to assemble all relevant data, both from the index study and from external sources, and then elicit subjective probability distributions from experts [31]. The sequence of events is summarised in Figure 3.

The study observations that will inform the elicitation will have been made in four hospitals; two where the IT includes advanced decision support, and two incorporating more rudimentary clinical algorithms. Eliciting probability densities for all four hospitals would be a tedious process. We will therefore elicit probabilities for just one high support hospital (selected at random), but the experts will be exposed to data from all hospitals. This will, we believe, provide an opportunity for nuanced data interpretation – for example if improvement is similar across high and low decision support hospitals, this will moderate cause and effect interpretations in the former group.

In line with good practice, the group from whom the densities will be elicited will be selected on the grounds that they are knowledgeable about the domain of enquiry but have no stake (emotional or other) in the results [32,33]. The expert members of the International Programme Steering Committee (IPSC) meet this requirement and we will therefore elicit probability densities from this constituency. Before attending for the elicitation exercise, participants will be sent a ‘dossier’ made up as follows:

1. Systematic review of salient evidence based on updated version of our previous review [8];

2. Results of the qualitative investigations in the four participating hospitals;

3. Before and after comparison of error rates across the four hospitals;

4. Before and after comparison of adverse event rates, both directly (but imprecisely) measured and modelled from the four selected error rates.

The group will discuss the above evidence and its limitations before taking part in the elicitation process. Discussion will be facilitated and the experts will discuss the above four data-types in series, before discussing what they may mean, and thereby synthesising evidence and argument. Probability densities will be elicited separately for each Hoonhout category. These probability densities will then be combined across experts.

The elicitation questionnaires have been informed by our previous experience [14,34,35], and are included as Additional file 2. In designing a questionnaire a number of decisions must be made [33]:

1) Whether to include a training exercise. In our case the respondents are familiar with Bayesian principles, so we have omitted this step.

2) Whether to ask respondents to assign probabilities to effect sizes of various magnitude (fixed interval) or to assign magnitudes corresponding to various probabilities (variable interval). Based on our previous experience we will use the first method only, not wishing to tire the experts (Additional file 2). The fixed interval method is often performed using the ‘chip and bin’ or ‘roulette’ method, which involves asking the expert to assign chips to various bins (into which the variable has been divided up) to build up their distribution of beliefs. Rather than using discrete chips we will ask the experts to mark a line to indicate the relative height of their density for that bin – a method that has worked well in the past (see figure in Additional file 2) [35].

3) Whether to elicit an effect size for the intervention (as Spiegelhalter has done) or separate estimates for control and intervention patients (as O’Hagan recommends and as we have used previously) [36]. The latter avoids the need to make assumptions about independence between baseline (control) rates and the intervention effect size, but we will select the former on the grounds that we have found it (anecdotally) to be more intuitive for clinicians, who are familiar with data presented in this way. We will, however, ask about percentage change (on a relative risk scale), which avoids experts having to think about small probabilities [37].

4) Whether to elicit individual subjective probability densities with a view to aggregating them or use a behavioural approach to aggregation and conduct a group elicitation [33]. We plan to use the first method, but the elicitation will be preceded by group discussion and an iterative process will be used, as described above.

5) Whether to use software or paper to record elicited data. We plan to replicate the data capture questionnaire on software to avoid the need for a two stage procedure.

After the questionnaires have been completed, we will pool the elicited distributions. We will then present the individual elicited (anonymised) probability distributions and the pooled probability distribution back to the group. In this way each member of the group will be able to reflect on their opinions and have a chance to revise them in the light of the opinions of other members of the group and the corresponding group consensus. Provided that permission is granted by all participants, the elicitation meetings will be video recorded for subsequent enquiry into the process of elicitation itself. A separate protocol will be written for this exercise.

### Assigning utilities

Utilities are not available for adverse events as a whole or in groups. They are seldom available for the individual events, death apart, unless they are naturally occurring disease states (such as deafness or kidney failure) that can also result from medication error. Moreover, it is the adverse event category for which we need adverse events in this study. We will obtain utilities through two separate methods:

1. We will select an archetypal example (Table 4) of an adverse event that may result from medication error within each category and for which utility estimates are available in the literature (severe gastro-intestinal bleed resulting from a prescription of non-steroidal anti-inflammatory drugs to a patient already on warfarin, for example).

2. We will elicit utilities for the Hoonhout groups (Table 4) using the time trade-off method [38]. We will again ask the members of the IPSC to take part in this exercise since we believe it would be very difficult for members of the general public to conceptualise groups of disease states. We cannot be certain that the people who are experts in the subject of medication error will find this exercise satisfactory, and it is for this reason that we include the first method above – it forms an insurance policy, as well as data for possible sensitivity analysis.

### Calculation of health benefit

When the above data have been collected the health benefit in Quality Adjust Life Years (QALYs) per hospital admission will be calculated as a sum over the four categories of the form:

$$\overset{4}{\underset{i=1}{\sum }}RR_{i}\times p_{i}\times U_{i}\times L_{i}$$

Where for each category (*i* = 1,2,3,4)

*RR*_*i*_ is the relative reduction in adverse event rate elicited from the expert group;

*p*_*i*_ is the pre-intervention prevalence of the event (i.e. overall prevalence x proportion from table 4);

*U*_*i*_ is the estimated loss of utility associated with the event;

*L*_*i*_ is the time period (in years) over which the loss of utility is experienced (Table 4).

The above calculation assumes that within the adverse event groupings, change in probability of event occurring, severity of event, and length of adverse event are all assumed to be independent.

### Determining net costs

There are three broad types of cost associated with HIT system:

1. Equipment costs (purchase and maintenance of hardware and software);

2. Training costs and effect of HIT use on staff time;

3. Costs contingent on changes in adverse event rates.

The first category above will be ascertained by document retrieval on site, backed up by interviews with vendors. Categories of staff time that may be affected (positively and negatively) by installation of an HIT system will be derived from qualitative interviews, and quantified by means of time and motion studies that will be described elsewhere. The third cost category will be calculated per patient using the formula: $\overset{4}{\underset{1}{\sum }}RR_{i}\times p_{i}\times C_{i}$

Where *C*_*i*_ is the cost of the adverse event class after Hoonhout et al. [28] The figures given by Hoonhout et al. will be converted from Euros to Pounds Sterling, converted for purchasing power parity through a Gross Domestic Product (GDP) Purchasing Power Parity (PPP) conversion factor [39], and updated to 2014 rates by applying the Hospital and Community Health Service (HCHS) Pay and Price Inflation Index (a weighted average of two separate inflation indices, the Pay Cost Index (PCI) and the Health Service Cost Index (HSCI).

### Calculation of cost-effectiveness

QALY gains will then be calculated for hospitals with 20,000, 35,000 and 50,000 admissions per year. Upfront hardware costs will be amortised over 20 years, applying a discount rate of 3.5%, in line with National Institute for Health and Care Excellence (NICE) guidance [40]. Annual costs of maintaining a computer system and employing staff will be added to the amortised capital expenditure. Cost savings from adverse events averted will be subtracted to yield a global net cost at the level of the health service (not individual hospital). This will enable us to calculate the incremental cost-effectiveness ratio (ICER):

$$\mathrm{ICER}\;=\;\left(\mathrm{Total}\;\mathrm{costs}\right)\;÷\;\left(\mathrm{Total}\;\mathrm{QALYs}\;\mathrm{gained}\right)$$

We will also express QALY gain as Expected Monetary Benefit (EMB):

$$\mathrm{EMB}\;=\;\left(\mathrm{QALYs}\;\mathrm{gained}\right)\;\times \;\lambda$$

Where λ is societal willingness to pay for one QALY – assumed to be £20,000 in the base case. This will also enable us to express the result as the Expected Net Benefit (ENB):

ENB=EMB−Δcosts

A problematic sensitivity analysis will then be performed by pooling the effectiveness distribution and a cost-effectiveness acceptability curve constructed to show the likelihood that the intervention is effective as a function of the threshold, including a zero threshold where it is cost-releasing.

## Discussion

### The epistemology of our proposed evaluation

The study is designed to deal with a frequent and justified criticism of many evaluations of information technology applications – namely that they do not, and cannot, capture all salient end-points [41]. An evaluation of this technology cannot ignore these end-points just because they cannot be captured objectively in numerical form. Health economic models require input parameters even if these cannot be measured directly; they must be assessed in some other way. We have previously approached this problem by capturing the necessary estimates in the form of a Bayesian probability distribution. In this study any observed reduction in errors and adverse events will be used to inform an elicited subjective estimate of the putative reduction in relative risk of adverse events as a whole, rather than to provide a direct estimate of that parameter.

Where a scenario can be described by a decision model, any pragmatic choice can be reverse-engineered into a subjective belief about the likely value(s) of some critical parameter or parameters. Consider a decision maker who wished to reduce adverse events. Choice of a prescribing system that claimed to have this effect would imply the decision maker believed the cost of the system was outweighed by health benefit associated with avoided events. But choices must frequently be made in the light of imperfect information about such parameters. Then it would be reasonable that a group of potential decision makers come together to discuss whether the system should be adopted, having weighed up all the pros and cons – i.e. all forms of evidence. This approach might well be applauded where no definitive objective answer could be obtained. What we envisage is to engage experts at a more basic level by unpicking their beliefs about the constituent parameters of a decision rather than their attitudes to the decision itself. Such beliefs, expressed as subjective probability densities, can then be combined with exogenous parameters (such as the cost-effectiveness threshold) to forward-engineer a rational approach to the decision itself in a particular policy environment.

### Modelling causal pathways to inform elicitation exercises

A rather unusual component of our protocol is the “calibration” method, whereby we propose modelling adverse events from just four error types. Two issues arise – whether such an exercise is helpful, and how, if helpful, examples should be selected. On the first point, our reading of the psychological literature is that methods that help the mind to decompose complex tasks are normative (mitigate heuristic biases). On the second point, we had much debate in committee over the selection of topics. We are aware of the potential criticism that errors associated with literature on potential harms may be a biased subset of the errors they are intended to represent. Depending on the size of this bias, this exercise could increase rather than mitigate bias. We would value feedback from the academic community on these points.

### Unresolved issues

One important limitation of the study is that it is based on health service costs and benefits, rather than a societal perspective, especially those resulting from permanent harm. The estimate of Hoonhout et al. of cost implications of adverse events took this narrower perspective, and also did not include cost consequences over the long term [28]. The model could be extended to take these longer term and broader societal impacts into account given the necessary parameter estimates. However, obtaining credible estimates for these parameters would be a research project in its own right. Unless such figures are published between now and publication of the results of our model, we plan to leave long-term benefits out of the model and simply qualify our results as conservative (i.e. a likely underestimate of cost-savings).

Any classification system is a compromise between detail and practicality. The system used by Hoonhout et al., to classify adverse events, conflates severity and duration, while that of Brennan et al. [17] and of Yao et al. [14] classify adverse events according to both dimensions (Table 3), producing six-point scales. However, we are mindful of the requirement to elicit both probabilities and utilities from our respondents and avoid elicitation fatigue. For this reason, and also because costs are available for it, we have proposed Hoonhout’s four-point scale, at least for the time being.

The wording of questions is important in eliciting probability densities. Service delivery interventions are context dependent [42] and it is therefore important to be clear about context in elicitation. We therefore make it clear that the context relates to those of the National Health Service (NHS) at the time of the intervention. A more controversial point concerns elicitation for just one of the four hospitals in the study of four cases. Certainly, to obtain separate distributions for each hospital would create elicitation fatigue, but densities could be elicited for groups of institutions – in this case adopters of high versus low level decision support. However, this risks lack of clarity about precisely what the parameter relates to, so our interim solution is to focus on a particular hospital. As in any research study, decision makers will need to exercise judgement when extrapolating across time and place.

## Conclusion

We present a method to deal with the “inconvenient truth” [5] that occurs when complex generic service delivery interventions must be assessed for cost-effectiveness. The method we propose here includes first, an assembly of relevant information on multiple end-points and contextual factors from within and outside of an index study. Instead of using this information to directly inform a decision, it is used to generate probability densities for the parameters of interest – in this case reductions in adverse events, by category, resulting from deployment of IT. A process of deliberative dialogues follows, so that experts can “re-calibrate” their subjective probability estimates in the light of, for example, factors they may have overlooked. A consolidated prior assembled from the repeat elicitation exercise can be used to populate a health economic model, along with salient utilities. The credible limits on these densities can define thresholds for sensitivity analyses.

### Ethics

The National Research Ethics Service (NRES) Committee London – City and East were consulted regarding ethical approval, and deemed a full ethical review by a NHS Research Ethics Committee unnecessary.

In line with basic ethical principles, we will ensure that all experts who undertake the elicitation questionnaire will participate voluntarily with informed consent and can withdraw from the study at any time.

## Abbreviations

EMB: Expected monetary benefit; ENB: Expected net benefit; GDP: Gross domestic product; HCHS: Hospital and community health service; HIT: Health information technology; HSCI: Health service cost index; HTA: Health technology assessment; ICER: Incremental cost effectiveness ratio; IPSC: International programme steering committee; NHS: National health service; NICE: National institute for health and care excellence; NIHR: National institute for health research; PCI: Pay cost index; PPP: Purchasing power parity; QALY: Quality adjusted life year.

## Competing interests

This cost-effectiveness analysis forms part of a National Institute for Health Research (NIHR) funded research programme to evaluate the implementation, adoption, effectiveness and cost-effectiveness of ePrescribing systems as they are introduced into a sample of hospitals in England (RP-PG-1209-10099). The authors declare no other competing interests.

## Authors’ contributions

RJL conceived the idea for the evaluation, and drafted the initial and subsequent core manuscripts; AJG advised on probability elicitation and methodology; AS is Principal Investigator on the NIHR Applied Programme Grant (RP-PG-1209-10099) and has overseen this, and related components of this programme of work; JJC was responsible for measurement of error and adverse events; PJC conducted work on the classification of adverse events and advised on the elicitation exercise; SLB advised on the calculation of health benefit and cost-effectiveness; DJJ advised on probability elicitation and methodology; LB conducted work on prescription errors and Additional file 1; KH advised on probability elicitation and the elicitation exercise. All authors critically reviewed and commented on several drafts of this manuscript, and read and approved the final manuscript.

## Pre-publication history

The pre-publication history for this paper can be accessed here:

http://www.biomedcentral.com/1472-6963/14/314/prepub

## Supplementary Material

Additional file 1 **Protocol for evaluation of the cost-effectiveness of ePrescribing systems.** Additional file 1. Key prescription errors that may be prevented using an electronic prescribing system. Click here for file

Additional file 2 **Protocol for evaluation of the cost-effectiveness of ePrescribing systems.** Additional file 2. Pro forma for elicitation of experts’ subjective probability densities. Click here for file
