# Supplementary material for: Protocol for evaluation of the cost-effectiveness of ePrescribing systems and candidate prototype for other related health information technologies
Source: BMC Health Serv Res. 2014 Jul 19;14:314. doi: 10.1186/1472-6963-14-314 (PMC4118257; doi:10.1186/1472-6963-14-314)
Supplement: Additional file 1 — Protocol for evaluation of the cost-effectiveness of ePrescribing systems. Additional file 1. Key prescription errors that may be prevented using an electronic prescribing system. [file 1472-6963-14-314-S1.docx]

**Additional file 1**

A national e-Delphi exercise has been carried out involving 20 experts to develop a consensus on key prescription errors that may be prevented using an electronic prescribing system. A list of 80 such errors was compiled, and a ranking for likelihood and seriousness of consequence assigned to each. A composite risk score for each error was then derived by taking the product of the rankings of each category. Appendix Table 1 shows the categories and definitions used in the process.

**Table S1**. Categories and definitions used.

| **Rank** | **Likelihood** | **Consequence** |
| --- | --- | --- |
| **1** | **Rare**  *This will probably never occur* | **Insignificant**  *No risk of patient injury or harm and no intervention required* |
| **2** | **Unlikely**  *Do not expect it to occur but it is possible it may do* | **Minor**  *Minor injury or illness requiring minor intervention* |
| **3** | **Possible**  *This might occasionally occur* | **Moderate**  *Moderate injury requiring intervention* |
| **4** | **Likely**  *This will probably occur* | **Major**  *Major injury leading to long-term incapacity/ disability* |
| **5** | **Almost certain**  *This will undoubtedly occur, possibly frequently* | **Catastrophic**  *Leads to death, multiple permanent injuries, or irreversible health effects* |

The errors considered divide broadly by type, with four main categories; drug-drug interaction, allergy, clinical contra-indication, and dosing. It is intended to select for modelling, a representative example from each of these categories. The adverse events associated with the sample of errors chosen will be divide by their nature and severity, and used to inform the larger aggregated economic model.

The potential benefits arising from the prevention of each of the 80 prescription errors depend on the frequency with which that error occurs and the nature of any consequential adverse outcomes. The frequency with which an error occurs depends on two factors, the number of occasions on which that prescription is indicated, and the probability that and error will then be made in prescribing. Likewise, the consequence of that error also has two components, the risk that a patient will suffer an adverse event given that a prescription error has been made, and the nature of that adverse event in terms of severity, duration, health-service costs, and loss of quality of life.

All of these factors need to be considered when selecting a representative sample of errors, and thus the decision is primarily driven by the relative importance attached to each of the elements of this causal chain. However, the selection will also be constrained by the availability of suitable evidence. It is clearly unethical to design a trial in which patients are deliberately given potentially dangerous treatment to mimic prescribing errors. Therefore, the evidence needs to be drawn from retrospective observational studies that link observed adverse events to prescription errors.
