# Supplementary material for: Protocol for evaluation of the cost-effectiveness of ePrescribing systems and candidate prototype for other related health information technologies
Source: BMC Health Serv Res. 2014 Jul 19;14:314. doi: 10.1186/1472-6963-14-314 (PMC4118257; doi:10.1186/1472-6963-14-314)
Supplement: Additional file 2 — Protocol for evaluation of the cost-effectiveness of ePrescribing systems. Additional file 2. Pro forma for elicitation of experts’ subjective probability densities. [file 1472-6963-14-314-S2.docx]

**Additional file 2**

**Bayesian Elicitation for the effectiveness of an ePrescribing system to
reduce prescription errors in English hospitals**

*About the study*

The National Institute for Health Research (NIHR) has funded a study to investigate the implementation, adoption, effectiveness and cost-effectiveness of ePrescribing systems as they are introduced into a sample of English hospitals (RP-PG-1209-10099).

A number of studies have demonstrated that prescribing errors are common in hospitals, and can lead to considerable morbidity and mortality. There is thus a need to identify effective methods to reducing prescribing error. One such potential method is the use of an ePrescribing system. However, ePrescribing systems vary considerably in terms of their functionality, inter-operability and costs. Furthermore, implementation of a comprehensive ePrescribing system is a ‘generic’ intervention that can potentially affect a large number of diffuse clinical processes (e.g. prescribing decisions) and outcomes (e.g. adverse events) to various degrees.

The study is comprised of:

1. Qualitative assessment of the effects of ePrescribing systems at the organisational level;
2. Measurement of prescribing safety in a before and after study in 4 hospitals;
3. Cost analysis;
4. Cost-effectiveness analysis.

This Bayesian elicitation is concerned with aiding in the analysis of the cost-effectiveness of such systems.

*About the cost-effectiveness analysis*

To analyse the cost-effectiveness of an ePrescribing system requires an estimate of its effectiveness in reducing adverse events; utilities for adverse events; costs associated with the system; and cost consequences associated with adverse events avoided. However, there is no single end-point that can adequately and unproblematically capture the effectiveness of such a complex intervention. Therefore although the study plans to observe changes in error rates and adverse events, we also plan to also elicit estimates of effectiveness from a group of experts following a group discussion, during which the results of systematic reviews of ePrescribing will be presented, along with qualitative and quantitative data consisting of:

- Results of the qualitative study, which will include a description of the systems;
- Before and after comparison of error rates across four hospitals (bearing in mind that this study has no contemporaneous controls);
- Before and after comparison of adverse event rates, both directly measured and modelled from the four selected error rates (bearing in mind that this comparison is very underpowered).

These expert opinions will then be pooled to create a probability distribution, assessing the effectiveness of the intervention from an inductive perspective.

*About the elicitation*

Prescribing errors can potentially cause a large number of different adverse events, which the intervention will affect to varying degrees. These events are also associated with various mean utilities. Therefore, we plan to classify adverse events according to their severity and duration, based on a classification system developed by Hoonhout et al. (2009 doi: 10.1186/1472-6963-9-27). Adverse events will thus be classified as having:

- Had minimal effect (2.5 per 1000 inpatients);
- Led to moderate disability; (6 per 1000)
- Led to permanent disability; (0.5 per 1000)
- Led to death. (1 per 1000)

*What might the effectiveness of this intervention be?*

For each category the participants will complete a questionnaire asking for their probabilities as to what the relative percentage changes will be.

*Example from previous study on effectiveness of laparoscopic uterosacral nerve ablation (LUNA):*

**
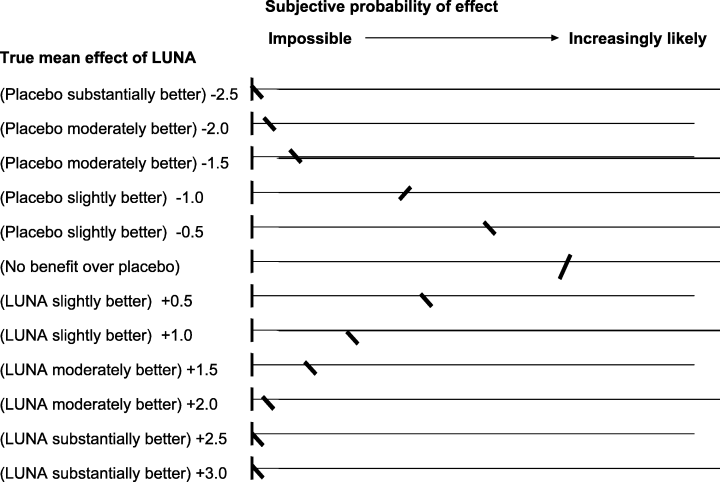
**

*Example of graphical elicitation of beliefs about the likely true effect of LUNA, compared with placebo (laparoscopy alone), in patients with chronic pelvic pain, as measured by change in 10-point visual analogue scale scores. (Latthe et al. 2004. doi:10.1111/j.1471-0528.2004.00304.x)*

This participant believed that, on average, there would be no benefit/harm of LUNA compared to placebo, and so marked the ‘No benefit’ line towards the right-hand side.

They believed that a disadvantage (i.e. placebo being better than LUNA) greater than 1.5 points would be highly unlikely, and so marked the -2.5, -2.0, -1.5 lines very close to the left-hand side.

Furthermore, they believed a mean benefit greater than 2.0 points would also be highly unlikely, and so marked the lines +2.0, +2.5, +3.0 towards the left-hand side.

The participant then considered how much less likely slight mean changes of ±0.5 would be compared to no benefit/harm, and marked the lines accordingly – i.e. that a small harm (-0.5) would be slightly less likely than no change, and a small benefit (+0.5) would be less likely than a small harm.

The remaining lines were marked using a similar thought process.

**MINIMAL IMPACT ADVERSE EVENTS**

Following discussion what is your opinion of the likely impact the ePrescribing intervention will have on **the rate of adverse events that have minimal impact on the patient** *(estimated to be about 2.5 per 1000 in patients at baseline)*?

1. **After the intervention period what do you think the rate of such events will be?**

|  | | | **Subjective probability of effect** |
| --- | --- | --- | --- |
| **Percentage Relative Reduction Change** | | | ***Impossible*** ------------------------------------------------------------🡪 ***Certain*** |
| Intervention is harmful | Increase in adverse events | 90 to 100% | \|------------------------------------------------------------------------------------------ |
|  | Increase in adverse events | 80 to 90% | \|------------------------------------------------------------------------------------------ |
|  | Increase in adverse events | 70 to 80% | \|------------------------------------------------------------------------------------------ |
|  | Increase in adverse events | 60 to 70% | \|------------------------------------------------------------------------------------------ |
|  | Increase in adverse events | 50 to 60% | \|------------------------------------------------------------------------------------------ |
|  | Increase in adverse events | 40 to 50% | \|------------------------------------------------------------------------------------------ |
|  | Increase in adverse events | 30 to 40% | \|------------------------------------------------------------------------------------------ |
|  | Increase in adverse events | 20 to 30% | \|------------------------------------------------------------------------------------------ |
|  | Increase in adverse events | 10 to 20% | \|------------------------------------------------------------------------------------------ |
|  | Increase in adverse events | 0 to 10% | \|------------------------------------------------------------------------------------------ |
| Intervention is beneficial | Decrease in adverse events | -10 to 0% | \|------------------------------------------------------------------------------------------ |
|  | Decrease in adverse events | -20 to -10% | \|------------------------------------------------------------------------------------------ |
|  | Decrease in adverse events | -30 to -20% | \|------------------------------------------------------------------------------------------ |
|  | Decrease in adverse events | -40 to -30% | \|------------------------------------------------------------------------------------------ |
|  | Decrease in adverse events | -50 to -40% | \|------------------------------------------------------------------------------------------ |
|  | Decrease in adverse events | -60 to -50% | \|------------------------------------------------------------------------------------------ |
|  | Decrease in adverse events | -70 to -60% | \|------------------------------------------------------------------------------------------ |
|  | Decrease in adverse events | -80 to -70% | \|------------------------------------------------------------------------------------------ |
|  | Decrease in adverse events | -90 to -80% | \|------------------------------------------------------------------------------------------ |
|  | Decrease in adverse events | -100 to -90% | \|------------------------------------------------------------------------------------------ |

*Please use the graph above to record your beliefs in more detail. Each horizontal line represents a possible true benefit/harm from the ePrescribing intervention compared to baseline. Mark each line with* ***a single line*** *to show how likely you believe that this level of benefit/harm is the true level.*

**MODERATE IMPACT ADVERSE EVENTS**

Following discussion what is your opinion of the likely impact the ePrescribing intervention will have on **the rate of adverse events that led to moderate disability of the patient** *(estimated to be about 6 per 1000 in patients at baseline)*?

1. **After the intervention period what do you think the rate of such events will be?**

|  | | | **Subjective probability of effect** |
| --- | --- | --- | --- |
| **Percentage Relative Reduction Change** | | | ***Impossible*** ------------------------------------------------------------🡪 ***Certain*** |
| Intervention is harmful | Increase in adverse events | 90 to 100% | \|------------------------------------------------------------------------------------------ |
|  | Increase in adverse events | 80 to 90% | \|------------------------------------------------------------------------------------------ |
|  | Increase in adverse events | 70 to 80% | \|------------------------------------------------------------------------------------------ |
|  | Increase in adverse events | 60 to 70% | \|------------------------------------------------------------------------------------------ |
|  | Increase in adverse events | 50 to 60% | \|------------------------------------------------------------------------------------------ |
|  | Increase in adverse events | 40 to 50% | \|------------------------------------------------------------------------------------------ |
|  | Increase in adverse events | 30 to 40% | \|------------------------------------------------------------------------------------------ |
|  | Increase in adverse events | 20 to 30% | \|------------------------------------------------------------------------------------------ |
|  | Increase in adverse events | 10 to 20% | \|------------------------------------------------------------------------------------------ |
|  | Increase in adverse events | 0 to 10% | \|------------------------------------------------------------------------------------------ |
| Intervention is beneficial | Decrease in adverse events | -10 to 0% | \|------------------------------------------------------------------------------------------ |
|  | Decrease in adverse events | -20 to -10% | \|------------------------------------------------------------------------------------------ |
|  | Decrease in adverse events | -30 to -20% | \|------------------------------------------------------------------------------------------ |
|  | Decrease in adverse events | -40 to -30% | \|------------------------------------------------------------------------------------------ |
|  | Decrease in adverse events | -50 to -40% | \|------------------------------------------------------------------------------------------ |
|  | Decrease in adverse events | -60 to -50% | \|------------------------------------------------------------------------------------------ |
|  | Decrease in adverse events | -70 to -60% | \|------------------------------------------------------------------------------------------ |
|  | Decrease in adverse events | -80 to -70% | \|------------------------------------------------------------------------------------------ |
|  | Decrease in adverse events | -90 to -80% | \|------------------------------------------------------------------------------------------ |
|  | Decrease in adverse events | -100 to -90% | \|------------------------------------------------------------------------------------------ |

*Please use the graph above to record your beliefs in more detail. Each horizontal line represents a possible true benefit/harm from the ePrescribing intervention compared to baseline. Mark each line with* ***a single line*** *to show how likely you believe that this level of benefit/harm is the true level.*

**SEVERE IMPACT ADVERSE EVENTS**

Following discussion what is your opinion of the likely impact the ePrescribing intervention will have on **the rate of adverse events that have led to permanent disability of the patient** *(estimated to be about 0.5 per 1000 in patients at baseline)*?

1. **After the intervention period what do you think the rate of such events will be?**

|  | | | **Subjective probability of effect** |
| --- | --- | --- | --- |
| **Percentage Relative Reduction Change** | | | ***Impossible*** ------------------------------------------------------------🡪 ***Certain*** |
| Intervention is harmful | Increase in adverse events | 90 to 100% | \|------------------------------------------------------------------------------------------ |
|  | Increase in adverse events | 80 to 90% | \|------------------------------------------------------------------------------------------ |
|  | Increase in adverse events | 70 to 80% | \|------------------------------------------------------------------------------------------ |
|  | Increase in adverse events | 60 to 70% | \|------------------------------------------------------------------------------------------ |
|  | Increase in adverse events | 50 to 60% | \|------------------------------------------------------------------------------------------ |
|  | Increase in adverse events | 40 to 50% | \|------------------------------------------------------------------------------------------ |
|  | Increase in adverse events | 30 to 40% | \|------------------------------------------------------------------------------------------ |
|  | Increase in adverse events | 20 to 30% | \|------------------------------------------------------------------------------------------ |
|  | Increase in adverse events | 10 to 20% | \|------------------------------------------------------------------------------------------ |
|  | Increase in adverse events | 0 to 10% | \|------------------------------------------------------------------------------------------ |
| Intervention is beneficial | Decrease in adverse events | -10 to 0% | \|------------------------------------------------------------------------------------------ |
|  | Decrease in adverse events | -20 to -10% | \|------------------------------------------------------------------------------------------ |
|  | Decrease in adverse events | -30 to -20% | \|------------------------------------------------------------------------------------------ |
|  | Decrease in adverse events | -40 to -30% | \|------------------------------------------------------------------------------------------ |
|  | Decrease in adverse events | -50 to -40% | \|------------------------------------------------------------------------------------------ |
|  | Decrease in adverse events | -60 to -50% | \|------------------------------------------------------------------------------------------ |
|  | Decrease in adverse events | -70 to -60% | \|------------------------------------------------------------------------------------------ |
|  | Decrease in adverse events | -80 to -70% | \|------------------------------------------------------------------------------------------ |
|  | Decrease in adverse events | -90 to -80% | \|------------------------------------------------------------------------------------------ |
|  | Decrease in adverse events | -100 to -90% | \|------------------------------------------------------------------------------------------ |

*Please use the graph above to record your beliefs in more detail. Each horizontal line represents a possible true benefit/harm from the ePrescribing intervention compared to baseline. Mark each line with* ***a single line*** *to show how likely you believe that this level of benefit/harm is the true level.*

**DEATH**

Following discussion what is your opinion of the likely impact the ePrescribing intervention will have on **the rate of adverse events that led to death of the patient** *(estimated to be about 2.5 per 1000 in patients at baseline)*?

1. **After the intervention period what do you think the rate of such events will be?**

|  | | | **Subjective probability of effect** |
| --- | --- | --- | --- |
| **Percentage Relative Reduction Change** | | | ***Impossible*** ------------------------------------------------------------🡪 ***Certain*** |
| Intervention is harmful | Increase in adverse events | 90 to 100% | \|------------------------------------------------------------------------------------------ |
|  | Increase in adverse events | 80 to 90% | \|------------------------------------------------------------------------------------------ |
|  | Increase in adverse events | 70 to 80% | \|------------------------------------------------------------------------------------------ |
|  | Increase in adverse events | 60 to 70% | \|------------------------------------------------------------------------------------------ |
|  | Increase in adverse events | 50 to 60% | \|------------------------------------------------------------------------------------------ |
|  | Increase in adverse events | 40 to 50% | \|------------------------------------------------------------------------------------------ |
|  | Increase in adverse events | 30 to 40% | \|------------------------------------------------------------------------------------------ |
|  | Increase in adverse events | 20 to 30% | \|------------------------------------------------------------------------------------------ |
|  | Increase in adverse events | 10 to 20% | \|------------------------------------------------------------------------------------------ |
|  | Increase in adverse events | 0 to 10% | \|------------------------------------------------------------------------------------------ |
| Intervention is beneficial | Decrease in adverse events | -10 to 0% | \|------------------------------------------------------------------------------------------ |
|  | Decrease in adverse events | -20 to -10% | \|------------------------------------------------------------------------------------------ |
|  | Decrease in adverse events | -30 to -20% | \|------------------------------------------------------------------------------------------ |
|  | Decrease in adverse events | -40 to -30% | \|------------------------------------------------------------------------------------------ |
|  | Decrease in adverse events | -50 to -40% | \|------------------------------------------------------------------------------------------ |
|  | Decrease in adverse events | -60 to -50% | \|------------------------------------------------------------------------------------------ |
|  | Decrease in adverse events | -70 to -60% | \|------------------------------------------------------------------------------------------ |
|  | Decrease in adverse events | -80 to -70% | \|------------------------------------------------------------------------------------------ |
|  | Decrease in adverse events | -90 to -80% | \|------------------------------------------------------------------------------------------ |
|  | Decrease in adverse events | -100 to -90% | \|------------------------------------------------------------------------------------------ |

*Please use the graph above to record your beliefs in more detail. Each horizontal line represents a possible true benefit/harm from the ePrescribing intervention compared to baseline. Mark each line with* ***a single line*** *to show how likely you believe that this level of benefit/harm is the true level.*

We would be grateful if you could also provide the following information:

| **Your Name** |  |
| --- | --- |
| **Organisation** |  |
| **Email** |  |
| **Telephone** |  |
| **Discipline** |  |
| **Title / position** |  |
